# Preparation of Carbon Dots@r-GO Nanocomposite with an Enhanced Pseudo-Capacitance

**DOI:** 10.3390/molecules28020541

**Published:** 2023-01-05

**Authors:** Qichen Liu, Kangkang Ge, Xiaoyan Wu, Zhiwei Zhu, Yu Zhu, Hong Bi

**Affiliations:** 1 School of Materials Science and Engineering, Anhui University, 111 Jiulong Road, Hefei 230601, China; 2 School of Chemistry and Chemical Engineering, Anhui University, 111 Jiulong Road, Hefei 230601, China

**Keywords:** carbon dots, pseudocapacitance, reduced graphene oxides, nitrogen-doping

## Abstract

Carbon materials with pseudocapacitive performance have attracted emerging interest in the energy storage and conversion field. Reduced graphene oxide (r-GO) with superior conductivity and electrochemical stability has been extensively investigated as an efficient capacitive electrode material. In this study, three-dimensional carbon dots (CDs)@r-GO hydrogel electrode was successfully in situ prepared by the one-pot method, where the CDs play a critical role in serving as both reduction agent and electrochemical active sites. With prolonged reaction time, the oxygen content of the CDs@r-GO nanocomposite material could be effectively reduced to ensure better electric conductivity, and the nitrogen content, which provides pseudocapacitance, was gradually increased. The representative two pairs of fast and reversible current peaks appeared in cyclic voltammetry curves, with around three times higher specific capacitance of CDs@r-GO hydrogel electrode (290 F g^−1^ at the current density of 1 A g^−1^ in 1 M H_2_SO_4_ electrolyte). This simple and mild approach is promising and it is believed it will shed more light on the preparation of high-efficiency and high-performance energy storage materials based on functional reductive CDs.

## 1. Introduction

Capacitive energy storage materials have attracted a tremendous amount of attention due to their fast charging and discharging rate and superior long-term cyclic stability. Limited by the specific capacitance of the electric double layer (EDLC) that originated from the physical charge separation mechanism, materials based on the EDLC mechanism, especially carbon materials, delivered a lower energy density (approximately 10 times lower than the battery materials). However, pseudocapacitive materials such as MnO_2_ and MXenes would bridge the gap of energy density between conventional EDLC capacitors and batteries while maintaining their superior rate performance [1,2]. Graphene is a kind of carbon nanomaterial with two-dimensional sp^2^ hybridized carbon frameworks that shows a larger theoretical specific surface area (2630 m^2^ g^−1^), excellent mechanical properties, high electrical conductivity (~10^7^ S m^−1^) and good electrochemical stability [3,4]. However, this superior performance would disappear when talking about a bulk electrode composed of graphene units. For instance, the capacitance of reduced graphene oxide (r-GO) prepared by traditional Hummer’s method is usually in the range of 100–200 F g^−1^, much lower than the theoretical value of 550 F g^−1^; this majorly results from the reduced specific surface area and the shortage of ion/electron transport rate [5,6,7,8]. Therefore, the three-dimensional r-GO hydrogel electrodes with thin nano-graphite walls that are composed of several layers of graphene are preferred. Normally, there are two routes to obtain r-GO hydrogel: thermal reduction in high-temperature and high-pressure environments, or a milder chemical reduction way [9,10,11,12]. Based on the well-prepared r-GO hydrogel, many efforts were carried out to introduce redox couples or functional surface groups to further improve their specific capacitance or, in another word, the energy density. For example, Zhong et al. explored the effect on the performance of graphene-based supercapacitors by N-doping with different phenylenediamines and they found that the introduction of nitrogen-based functional groups could increase the specific capacitance to 645 F g^−1^ [13]. Duan et al. doubled the specific capacitance by modifying the graphene materials with hydroquinone functional groups [14]. However, although a great process has been obtained for preparing the pseudocapacitive carbon nanomaterials, it still leaves plenty of space for further optimizing the experimental route and improving the total electrochemical performance.

Carbon dots (CDs) are a kind of zero-dimensional spherical carbon material with carbonized core inside and functional groups on their surface, which is usually obtained by polymerization and carbonization of organic small molecules. Due to their small size, good electric conductivity, water solubility and abundant functional groups on the surface, carbon dots were considered suitable additives or decorations to further improve the performance of the composite energy storage materials. [15,16,17,18] For instance, Li et al. synthesized graphite CQDs using an improved chemical oxidation method and used it as an intercalator to prepare a novel three-dimensional N, P co-doped CQDs/reduced graphene oxide (rGO) composite aerogel (N, p-CQDs/rGO) by a one-step hydrothermal method. Due to the synergistic effect of N and P and the introduction of CQDs, the performance of the supercapacitor is greatly improved [11]. Zhu et al. improved the electrochemical performance of graphene composite aerogels by using carbon quantum dots (CQDs) as a conductor, intercalators and stable links [12]. In terms of sodium-ion batteries, Kim et al. successfully prepared an LTO/N-doped graphene quantum dots (N-GQDs) composite material in a sodium-ion battery, where N-GQDs can simultaneously improve the electronic conductivity and ion transport rate. This endows the material with better rate performance and cyclic stability [19]. Despite the great progress mentioned above, the reductive ability of carbon dots was rarely reported in the field of energy storage materials. It is common to use the reductive property of CD, for sensor applications where the Ag^+^ will be reduced into Ag nanoparticles on the surface of CDs [20,21].

**Figure 1:**
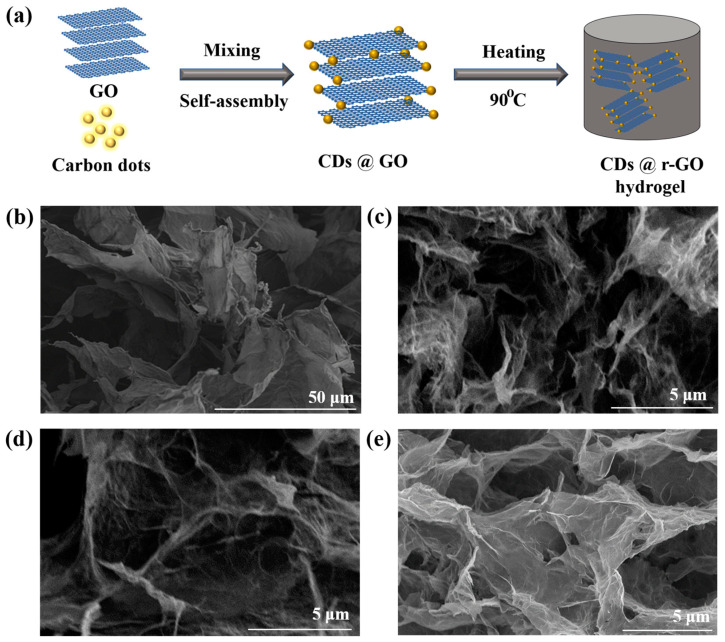
(**a**) Illustration of preparing CDs@r-GO composite materials; Scanning electron microscope images of (**b**) GO, (**c**) CDs@r-GO-1, (**d**) CDs@r-GO-4 and (**e**) CDs@r-GO-6.

Here, we first report the in situ preparation of CDs@r-GO hydrogel through a mild and simple one-pot synthesis with the help of the reductive, water-soluble and conductive carbon dots, as shown in Figure 1a. The CDs prepared from nitrogen-containing precursors exhibit reductive ability, which will serve as a reduction agent of GO in solution, and lead to the formation of r-GO hydrogel via a low-temperature heating (below 100 °C) process and within a short reaction time (6 h). The CDs doped with nitrogen would introduce more electrochemical active sites for energy storage and three-dimensional r-GO hydrogel with porous structure provides both the ion transports channel and electron transfer frameworks [22,23]. Therefore, the obtained CDs@r-GO composite hydrogel is believed to simultaneously improve the specific capacitance and maintain an excellent rate performance at the same time.

## 2. Results and Discussion

The nitrogen-doped CDs were synthesized from the nitrogen-containing o-phenylenediamine and EDTA-Na, Mn precursors. The hydrophilic oxygen-containing surface groups such as -COOH and -OH make CDs highly soluble in water, which also endows the following preparation process easier and safer. The average particle size of the CDs is around 1.95 nm (Supplementary Materials Figure S1). Then, GO prepared from the modified Hummer’s method was mixed with CDs; we assume that the CDs were adsorbed to the surface of GO in the solution, as we found that the strength of the fluorescence of the solution gradually decreased until it disappeared with the addition of GO dispersions (Figure S2). Fluorescence spectrometric analysis showed that the CDs had emission peaks at 290 nm and 550 nm, respectively. The corresponding emission of 290 nm originated from the core of CDs, while the visible yellow emission of 550 nm originated from the surface of CDs (Figure S3) [24]. The fluorescence intensity of the composite CDs@r-GO decreases for both the two emission peaks and this is another evidence to say the combination of CDs and GO—results in a change in the electronic structure of the composite material. Moreover, the fluorescence of the mixed solution after filtration is also indiscernible, which also demonstrates the successful combination of CDs and r-GO. The zeta potential of CDs and r-GO are −24 mV and −13 mV, respectively, which is attributed to the hydrolysis of -COOH or -OH surface groups. Normally, anchoring the negatively charged CDs into the same negatively charged r-GO is not easy [25,26]; there should be a specific attractive interaction that goes beyond the repulsive interaction between CDs and r-GO in our case. Then, the mixed solution with GO and CDs was heated at 90 °C to obtain r-GO hydrogel with CDs incorporated inside [13,27]. Here we define the obtained hydrogel as CDs@r-GO-1, CDs@r-GO-4 and CDs@r-GO-6, as the heating lasted for 1 h, 4 h and 6 h, respectively. The color of the solution changed from brown to transparent, which means approximately the end of the reaction without residual GO left in the solution. After lyophilization of the obtained hydrogel, dried CDs@r-GO aerogel was obtained. For structure analysis, GO demonstrated a large two-dimensional layered structure from the scanning electron microscope (SEM) image (Figure 1b). Moreover, the dried CDs@r-GO aerogel all exhibited obvious macro-porous structure, and the diameter of those pores extended from tens of nanometers to several microns (Figure 1c–e) [13,27]. These porous structures are highly favorable for electrolyte infiltration and are thus good for ion transport. They are expected to increase the effective contacting area between electrolyte and r-GO electrodes, resulting in an enhanced EDLC capacitance with lower resistance as well.

Moreover, the three-dimensional connecting carbon walls that are composed of several layers of graphene provide the mechanical strength and the electron transfer channel. When reaction time was extended, their structure remained without obvious change from SEM images, and it is similar to the morphology of r-GO obtained by sodium ascorbate reduction (conventional chemical reduction method to prepare r-GO hydrogel) (Figure S4).

**Figure 2:**
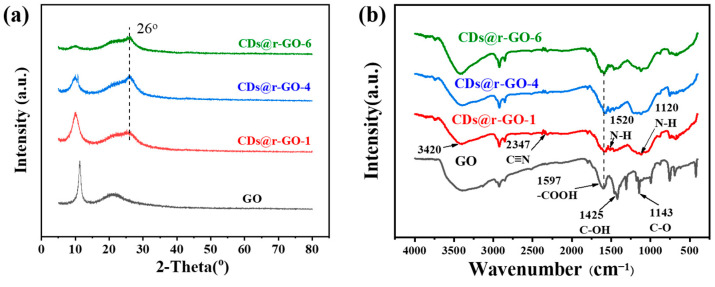
(**a**) X-ray diffraction patterns and (**b**) Fourier-transform infrared spectra of GO, CDs@r-GO-1, CDs@r-GO-4 and CDs@r-GO-6.

To further analyze the crystalline structure, powder X-ray diffraction (XRD) is carried out (Figure 2a). By comparing the XRD patterns, we found a regular and continuous shift of the diffraction peak with increasing reaction time. The diffraction peak at 11° of GO is attributed to the highly ordered (002) crystal plane. The intensity of this characteristic diffraction peak of GO gradually decreases with increasing time until it is almost negligible at the 6 h mark, which means the short-range order of GO layers was lost. Interestingly, a new diffraction peak at 26° appeared even at very early reaction stage (CDs@r-GO-1), which is referred to the characteristic (002) crystal plane of graphene-based materials, with a lattice distance of 0.34 nm [28]. The sp^2^ hybridized graphene structure was formed at an early stage and retained until the end of the preparation of this composite material. The extended reaction time facilitated the reduction of GO, which is accompanied by the destruction of the short-range order of GO [10]. The Fourier-transform infrared spectroscopy (FT-IR) provides more details for this reduction reaction. As shown in Figure 2b, there are a lot of oxygen-containing functional groups [29], the absorption peak at wave numbers of 1143 cm^−^^1^ corresponds to the vibration mode of C-O, 1425 cm^−^^1^ corresponds to the vibration mode of C-OH, and 1597 cm^−^^1^ indicates the vibration peak of -COOH [30,31]. After the reaction, a new band emerges, for example, the band at 1120 cm^−1^ together with the bands at 3420 cm^−1^ and 1520 cm^−1^ were assigned to N-H bending vibrations. Moreover, the bands at 1597 cm^−1^ and 2347 cm^−1^ were assigned to C=N and C≡N vibrations. That means that the N-containing functional groups of CDs@r-GO composite materials may come from the addition of N-doping CDs, and they remained even after extending the reaction time to 6 h [32]. Those oxygen-containing functional groups all disappeared after CDs addition and heated for hours. The removal of oxygen-containing surface groups resulted from the reductive properties of CDs as prepared, therefore the mechanism of formation of r-GO hydrogel should be similar to conventional chemical reduction process, as usually triggered by small reductive molecules such as sodium ascorbate [10,33,34]. In addition, as shown in Figure S5, we found two peaks at 1345 cm^−^^1^ and 1587 cm^−^^1^, corresponding to the D and G bands, respectively. By comparing the intensity values of the two peaks, it was found that I_D_/I_G_ gradually increased from 0.91 to 1.04 with the increase of reaction time, and the introduction of carbon dots and heating resulted in the increase of graphene defects [12]. To further make sure that it was the CDs themselves that served as the reduction agent, a comparative experiment was carried out: the pure GO dispersion without CDs addition was heated at the same conditions (90 °C, 6 h) It is called GO-90 °C-6 h. We found that the color of the solution remained brown, which is the color of GO dispersion. Moreover, we did not find a major change of the elements including C, N and O before and after heating (Table S1). In addition, we compared the XPS C1s narrow spectrum of GO-90 °C-6 h and GO, we found that their spectral shapes did not change significantly, indicating that only heating GO at 90 °C for 6 h could not play a reducing role (Figure S6). 

To further characterize the content change of main element of composite materials. XPS data were collected and was shown in Table 1. There was the highest oxygen content: 24.4 wt.% of GO, which mainly comes from the surface functional groups as discussed above. The O-containing groups were removed after addition of CDs and the oxygen content gradually decreased from 24.4 wt.% to 5.3 wt.% when the reaction time was extended to 6 h. Of course, the carbon content gradually increased, from 73.0 wt.% to 86.2 wt.%. As revealed by XRD, the more ordered graphitized carbon framework, together with the substantial loss of oxygen groups, would greatly improve the electric conductivity of the r-GO with reduced charge transfer resistance [35]. In addition, the content of nitrogen (N) increased gradually with increasing of reaction time and the only source of N doping should be the CDs. Normally, N, P and S are commonly used doping elements to enhance the conductivity and the specific capacitance of carbon-based pseudocapacitive electrode materials [36,37,38,39,40]. Here, it is good to have an increased N doping of the composite materials with longer reaction time.

**Figure 3:**
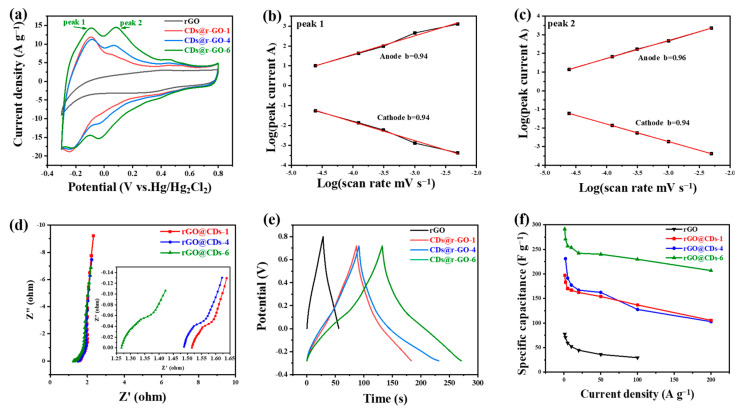
Electrochemical performance in 1 M H_2_SO_4_ electrolyte and in a three-electrode configuration. (**a**) Cyclic voltammetry curves at a scan rate of 50 mV s^−1^; (**b**,**c**) The plots of log (peak current) versus log (scan rate); (**d**) Nyquist plots; (**e**) Galvanostatic charge and discharge curves at a current density of 2 A g^−1^; (**f**) Rate performance at different charge and discharge current densities of r-GO, CDs@r-GO-1, CDs@r-GO-4 and CDs@r-GO-6.

Then the electrochemical energy storage performance of the composite materials was evaluated in 1 M H_2_SO_4_ electrolyte and in a three-electrode configuration. The pure r-GO was prepared for comparison, which is obtained by the conventional chemical reduction method; the detailed process can be found in the Section 3.3 and Section 3.4 of Materials and Methods. According to the cyclic voltammetry (CV) curve (Figure 3a), r-GO demonstrates a rectangular CV shape, in accordance with the feature of the capacitive energy storage process. There was a little distortion of the rectangular CV curve of r-GO at cathodic polarization, which was normal and was mainly limited by the electrolyte decomposition window that was controlled by the hydrogen evolution process in the situation of our acidic electrolyte. However, the shape of the CV curves of CDs@r-GO was totally different. On the one hand, two pairs of redox peaks of CDs@r-GO emerged with a greatly enlarged CV area which is proportional to the energy density. The two pairs of reversible redox reactions would provide considerable pseudocapacitance and, in another word, energy densities. On the other hand, the potential window was also extended when compared with pure r-GO, which means that CDs@r-GO-6 can store more energy and have a larger specific capacitance.

As the current of CV is controlled by the mechanism of redox reactions, the capacitive energy storage process (including EDLC and pseudocapacitive energy storage) delivered a relationship: current is proportional to the scan rate. On the other hand, for the battery-type material, the current is proportional to the square root of the scan rate. The current response of the CV curves at different scan rates depends on the charge storage mechanism according to the equation *i* = *av*^*b*^, where *i* is the peak current at different scan rates (*v*), and *a* and *b* are constants. The battery-type charge storage mechanism makes *b* = 0.5 while the capacitive charging process makes *b* = 1. Therefore, we plotted log(peak current) versus log(scan rate) to obtain the slope to evaluate the kinetic performance of the two redox reactions, and the CV with different scan rates are shown in Figure S7. The slopes for peak 1 are 0.94 for both cathodic and anodic peaks, and the slopes are 0.96 for anodic and 0.94 for cathodic (Figure 3b,c). Those slope values are highly close to 1.00, which means that the two pairs of redox reactions are fast, and their kinetics are mainly controlled by the surface reaction rather than the diffusion process. Therefore, it is believed to have an excellent rate performance on the condition of high-rate charging and discharging [41,42]. The electrochemical impedance spectroscopy (Figure 3d) shows that CDs@r-GO-6 has the smallest contact resistance for only 1.26 Ω. This value could be read from the intersection of Nyquist plots and the real part of impedance. This small contact resistance of CDs@r-GO-6 may be related to the highest nitrogen content, and nitrogen doping would improve the surface infinity and conductivity as well. The better wettability with aqueous electrolyte and good electric conductivity can reduce the contact resistance [36]. According to the galvanostatic charge-discharge files of CDs@r-GO at the current density of 2 A g^−1^ (Figure 3e), the CDs@r-GO-6 sample has the longest discharge time, which means that the specific capacitance is the largest. The calculated specific capacitance at the current density of 2 A g^−1^ is 271 F g^−1^, which is 3.8 times larger than the pure r-GO electrodes (71 F g^−1^). Moreover, the specific capacitance of r-GO, CDs@r-GO-1, CDs@r-GO-4 and CDs@r-GO-6 were calculated at different current densities (Figure 3f). As shown in Figure S8, at each current density that ranges from 1 A g^−1^ to 100 A g^−1^, the specific capacitance of CDs@r-GO-6 is highest. Furthermore, the rate performance of CDs@r-GO-6 was also the most excellent. When the current density increases from 2 A g^−1^ to 100 A g^−1^, the specific capacitance of CDs@r-GO-6 falls from 271 F g^−1^ to 230 F g^−1^ with a capacitance retention of 85%, whereas the capacitance retention rate of CDs@r-GO-1 and CDs@r-GO-4 is only 72% and 67%, respectively. The impressive rate performance and specific capacitance of CDs@r-GO-6 at 100 A g^−1^ are superior among the graphene-based carbon electrode materials (Table S2). From the electrochemical point of view, there are some reasons accounting for this surprising improvement: a lower interfacial resistance as revealed by Nyquist plots and the fast, reversible redox reactions facilitate the high-rate charging and discharging process. We used CDs@r-GO-6 as the negative electrode material and porous activated carbon as the positive electrode material to make the CR2032-type coin cell with 1M H_2_SO_4_ as the electrolyte, as shown in Figure S9. It shows a rectangular CV shape with good capacitive behavior; the shape of CV remains even after 15,000 cycles (Figure S9a). According to the GCD curve (Figure S9b), it also shows no big decay after 15,000 cycles, which means that the CDs@r-GO-6 as active materials have a good CV performance and cyclic stability at the device level [32].

**Figure 4:**
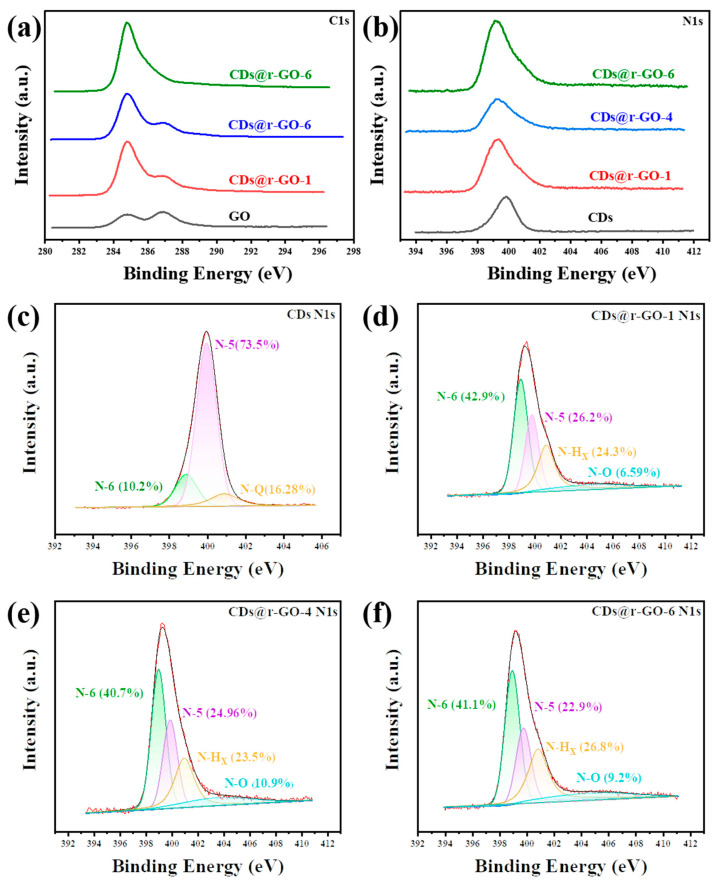
(**a**) XPS C1s spectra and (**b**) N1s spectra of the GO and CDs@GO composite materials. XPS N1s spectra of (**c**) CDs, (**d**) CDs@r-GO-1, (**e**) CDs@r-GO-4 and (**f**) CDs@r-GO-6.

To investigate the molecular origin of the superior electrochemical performance of the CDs and r-GO composite material, the chemical information was extracted through X-ray photoelectron spectroscopy (XPS). Figure 4a shows the high-resolution C1s XPS spectra for both the GO and obtained composite material. We found two types of carbon bonding states: one is the classic C-C bonding that is located at about 285 eV, and the other one corresponds to the C-O bonding at GO surface, which is located at about 287 eV [26]. With the increase of reaction time, the peak at 287 eV gradually decreases, which means the removal of C-O functional groups. Moreover, the integrity of carbon framework as revealed by C1s XPS spectra also pointed out that the obtained material has an excellent electron transport rate with increasing reaction time. This partly accounts for the superior rate performance of the composite material. Moreover, the high-resolution N 1s spectra (Figure 4b–f) provide how the reaction occurs and what an important role the doped N played in constructing excellent energy storage materials. The location of the N 1s peak gradually shifted to the lower binding energy by increasing the reaction time. It is about 0.8 eV shift of CDs@r-GO-6 when compared to the original GO. This indicates that N in CDs lost electrons in the reaction process, which again proves that CDs served as a reduction agent in preparing the CDs and r-GO composite material.

Furthermore, the N 1s spectra were deconvoluted to obtain the information on various types of N-based species. As shown in Figure 4c, there are three types of N existed in CDs: Pyridinic-N (N-6), Pyrrolic-N (N-5) and Quaternary-N (N-Q). The high content of Pyrrolic-N (N-5) (73.5%) of CDs would introduce a lot of redox-active sites for composite materials, as the N-5 greatly contributes to the pseudocapacitance [43,44]. As for the composite materials, nitrogen oxide (N-O) appears, which also provides redox-active sites for the electrochemical energy storage process [45]. Therefore, the N-5 together with N-O promote the rapid diffusion and transportation of ions and increase the fast and reversible faradic pseudo-capacitance, while the N-6 and N-Q can remarkably enhance the electronic conductivity and improve the rate capability of the composite materials [46,47]. Moreover, N-6 and N-Q can also effectively improve the wettability of the electrode and facilitate electrolyte infiltration [48,49]. The higher content of N-doping and rich types of N contribute to the improved performance of composite materials when compared with pure r-GO [50].

## 3. Materials and Methods

### 3.1. Materials

The materials include graphene oxide (GO, Gaoxi Tech, Hangzhou, China), ethylenediamine tetraacetic acid disodium manganese (AR, 95%, Macklin, Shanghai, China), o-phenylenediamine (OPD, AR, 98%, Macklin, Shanghai, China) and deionized water (H_2_O was prepared by a water purification system with a resistance of 18.2 MΩ cm).

### 3.2. Preparation of Carbon Dots (CDs)

A total of 0.44 g manganese disodium ethylenediamine tetraacetic acid (EDTA-Mn, Na) and 0.20 g o-phenylenediamine were dispersed and dissolved in 30 mL water, then put into the high-pressure reactor and heated inside Muffle furnace at 180 °C for 6 h. The yellow CDs solution was filtered and lyophilized to obtain CDs powder.

### 3.3. Preparation of r-GO

Firstly, 20 mg of GO was dispersed in 10 mL of deionized water, and then 40 mg of sodium ascorbate was added to the GO dispersion solution and followed by ultrasonication for 10 min. Then, the r-GO hydrogel was obtained by heating the mixed solution at 90 °C for 1.5 h. The obtained r-GO hydrogel was dialyzed against deionized water for 3 days to remove unreacted sodium ascorbate and other impurities. Then the r-GO aerogel could be obtained by freeze-drying.

### 3.4. Preparation of CDs @ r-GO Composite Material

Firstly, 40 mg CDs were dissolved in 8 mL of water, and then 2 mL (10 mg mL^−1^) GO dispersion was added into the solution drop by drop. The solution was sonicated for 15 min and stirred continuously to make the GO dispersion uniform. The mixed solution was heated at 90 °C for 1 h, 4 h and 6 h, respectively. The obtained hydrogel was dialyzed against deionized water for 3 days to remove unreacted sodium ascorbate and other impurities. Then the CDs@r-GO aerogel could be obtained by freeze-drying.

### 3.5. Characterization

The morphology and composition of the CDs@r-GO microstructure were determined by the transmission electron microscopy (TEM, JEM2100, JEOLLtd., Tokyo, Japan) and the scanning electron microscope (SEM, S-4800, Hitachi, Ltd, Tokyo, Japan). The elemental information was characterized by the X-ray photoelectron spectroscopy (XPS, ESCALAB250, Thermo Fisher, MA, Waltham, USA). Surface functional groups of CDs@r-GO were detected by the Fourier transform infrared (FT-IR, Vertex80 and Hyperion 2000, Bruker, Karlsruhe, Germany). Powder XRD analyses were carried out on an X’Pert PRO diffractometer with Cu Kα radiation (λ = 1.54056 Å) at a scanning rate of 20° min^−1^. PL spectra were recorded using a fluorescence spectrophotometer (F-4500, Hitachi, Ltd, Tokyo, Japan).

All electrochemical experiments were carried out on a CHI 660E electrochemical workstation. The materials-coated carbon cloth served as the working electrode, Hg/Hg_2_Cl_2_ (in saturated KCl) as the reference electrode, and porous activated carbon electrode as the counter electrode, respectively. The electrochemical performance of the as-prepared samples was characterized by the CV test and GCD test in a three-electrode system and the potential windows were set in the range from −0.3 to 0.7 V. The working electrode was prepared by first mixing the as-synthesized composite material (namely, carbon dots@r-GO) with the conductive carbon (acetylene black) and the binder (polyvinylidene difluoride: PVDF) in the weight percent ratio of 80:10:10, respectively. Then, a slurry consisting of the above mixture in a dropwise manner to form a homogeneous slurry was smeared onto a piece of carbon cloth (1 × 1 cm^2^) with a loading of 1.0 mg cm^−2^. Finally, the slurry was dried at 60 °C in vacuum for 12 h. All of the as-prepared electrodes were dipped into 1 M H_2_SO_4_ aqueous solution electrolyte.

### 3.6. Assembly of Supercapacitor Devices

The negative electrode was prepared by first mixing the as-synthesized composite material (namely, carbon dots@r-GO) with the conductive carbon (acetylene black) and the binder (polyvinylidene difluoride: PVDF) in the weight percent ratio of 80:10:10, respectively. The positive material was prepared by mixing porous activated carbon, conductive carbon black (acetylene black) and binder (polyvinylidene difluoride: PVDF) in a ratio of 80:10:10. The device was fabricated in a CR2032-type coin cell with carbon cloth served as the current collector, and 1M H_2_SO_4_ as the electrolyte. The load mass of CDs-r-GO-6 is about 0.8 mg cm^−2^, and that of porous activated carbon electrode is 2.16 mg cm^−2^, in order to match the charge balance of the positive and negative electrodes.

## 4. Conclusions

In conclusion, we reported a mild one-pot preparation of carbon dots and reduced graphene oxide composite hydrogel electrodes. In our case, the nitrogen active sites provide both the reductive ability for GO reduction and redox-active sites for efficient and high-performance energy storage. It provides an efficient way to combine two kinds of repulsive particles and improve their overall performance as well. The obtained CDs@r-GO materials with graphitized three-dimensional porous structure delivered a higher specific capacitance of 271 F g^−1^ at 2 A g^−1^ and an excellent rate performance with 230 F g^−1^ at 100 A g^−1^ (only ~20% decay of capacitance when current density increased from 2 A g^−1^ to 100 A g^−1^). This preparation process avoids strict conditions, such as high temperature, and is not time-consuming. In contrast, it is cost-effective, environmentally friendly and highly efficient. It will shed more light on the preparation of high-performance pseudocapacitive energy storage materials.

## Figures and Tables

**Table 1:** Analysis of elemental contents obtained by XPS.

Samples	C (wt.%)	O (wt.%)	N (wt.%)
GO	73.0	24.4	2.6
CDs@r-GO-1	80.2	15.5	4.3
CDs@r-GO-4	85.1	9.1	5.8
CDs@r-GO-6	86.2	5.3	8.5

## Data Availability

The data presented in this study are available on request from the corresponding author.

## Supplementary Materials

The following supporting information can be downloaded at: https://www.mdpi.com/article/10.3390/molecules28020541/s1, Figure S1. (a) High resolution TEM image and (b) the size distribution of CDs; Figure S2. Photo of the solution including under UV irradiation when gradually adding GO into the CDs solution; Figure S3. The fluorescence spectra of CDs, GO and CDs@GO (a) the excitation wavelength is 245 nm (b) the excitation wavelength is 490 nm; Figure S4. Scanning electron microscope image of r-GO obtained by sodium ascorbate reduction method; Figure S5. The Raman spectrum of GO, CDs@r-GO-1, CDs@r-GO-4 and CDs@r-GO-6; Figure S6. XPS C1s narrow spectrum of the GO and GO-90 °C-6 h; Figure S7. Cyclic voltammetry of (a) r-GO, (b) CDs@r-GO-1, (c) CDs@r-GO-4 and (d) CDs@r-GO-6 in 1 M H_2_SO_4_ electrolyte with scan rate ranging from 10 to 100 mV s^−1^; Figure S8. (a) Constant current charge and discharge curve of the r-GO (current density 1–100 A g^−1^) (b) Constant current charge and discharge curve of the CDs@r-GO-1 (current density 1–100 A g^−1^) (c) Constant current charge and discharge curve of the CDs@r-GO-4 (current density 2–100 A g^−1^) (d) Constant current charge and discharge curve of the CDs@r-GO-6 (current density 1–100 A g^−1^); Figure S9. (a) Cyclic voltammetry curve of the device (Scan rate 50 mV s^−1^) (b) Constant current charge and discharge curve of the device (current density 1 A g^−1^) (c) The photo of device fabricated; Table S1. Analysis of element content obtained by XPS test; Table S2. Comparison with data of electrode materials in other references.

## Sample Availability

Samples of the compounds are not available from the authors.
