# Supplementary material for: Preparation of Carbon Dots@r-GO Nanocomposite with an Enhanced Pseudo-Capacitance
Source: Molecules. 2023 Jan 5;28(2):541. doi: 10.3390/molecules28020541 (PMC9865864; doi:10.3390/molecules28020541)
Supplement: Supplementary file 1 [file molecules-28-00541-s001.zip › molecules-2145795-supplementary.pdf]

# Supporting Information

## Preparation of carbon dots@r-GO nanocomposite with an enhanced pseudo-capacitance

**Qichen Liu[1], Kangkang Ge[2], Xiaoyan Wu[1], Zhiwei Zhu[2], Yu Zhu[2], Hong Bi[1,*]**

[1]School of Materials Science and Engineering, Anhui University, 111 Jiulong Road, Hefei 230601, China

[2]School of Chemistry and Chemical Engineering, Anhui University, 111 Jiulong Road, Hefei 230601, China

*Correspondence: bihong@ahu.edu.cn

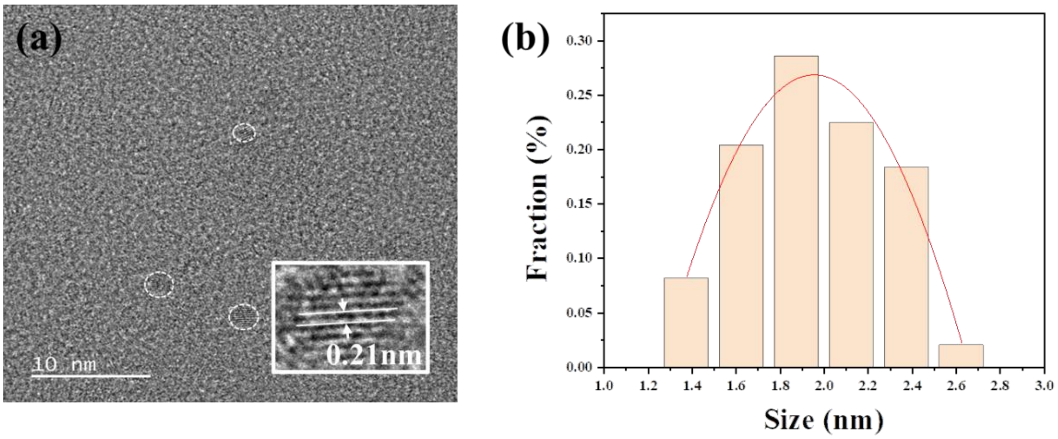


**Figure S1. (a)** High resolution TEM image and **(b)** the size distribution of CDs.

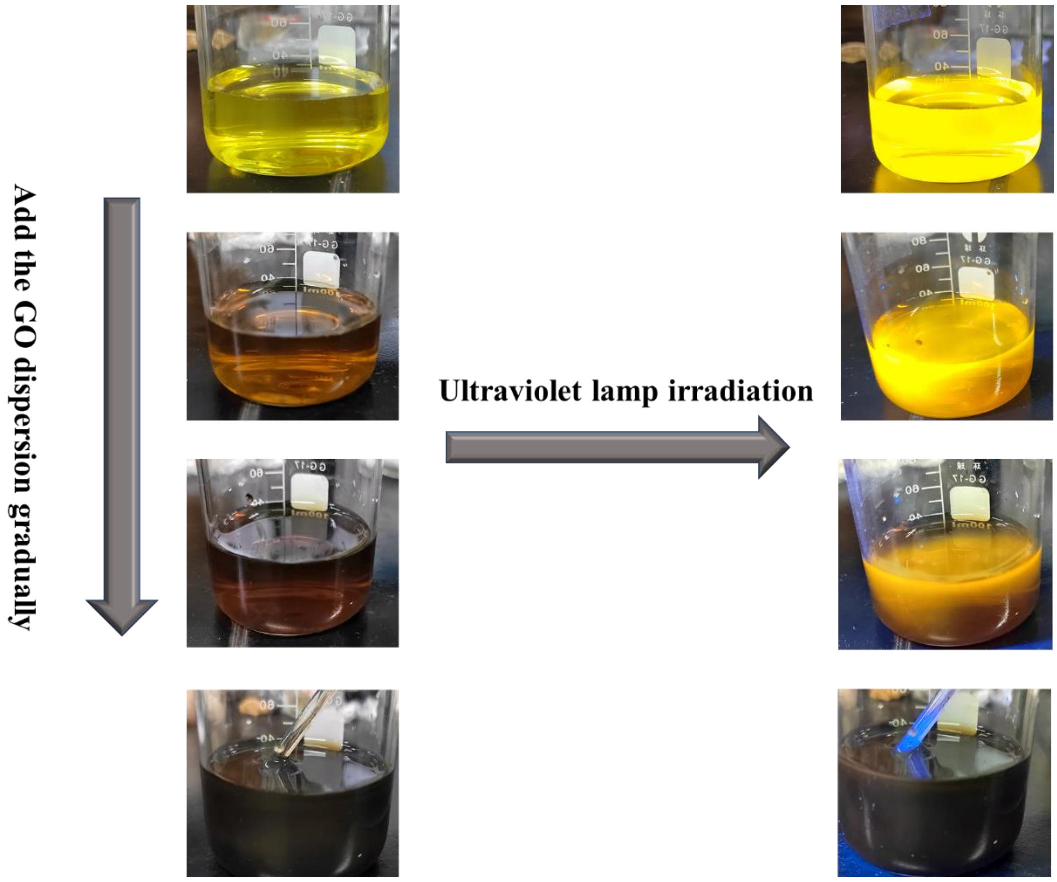


**Figure S2.** Photos of the solution containing the CDs under UV irradiation when gradually adding GO into the solution.

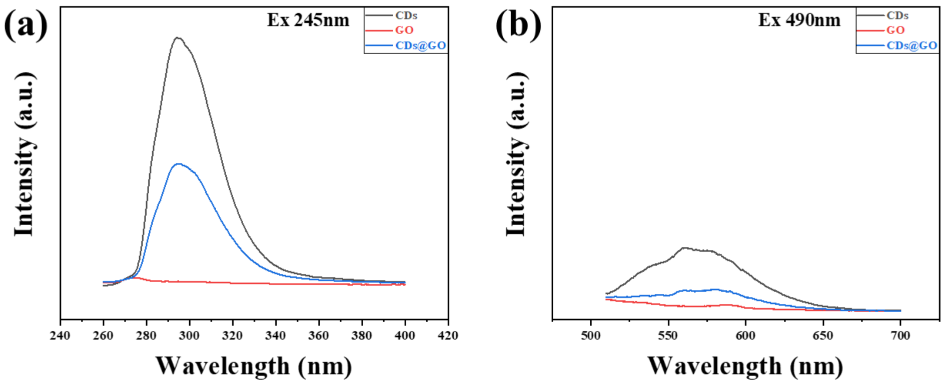


**Figure S3.** PL spectra of the CDs, GO and CDs@GO at the excitation wavelength of **(a)** 245 nm and **(b)** 490 nm.

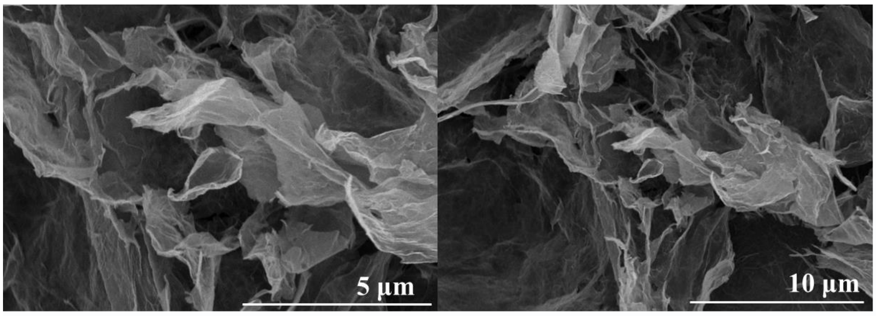


**Figure S4.** Scanning electron microscope (SEM) images of r-GO obtained by sodium ascorbate reduction method.

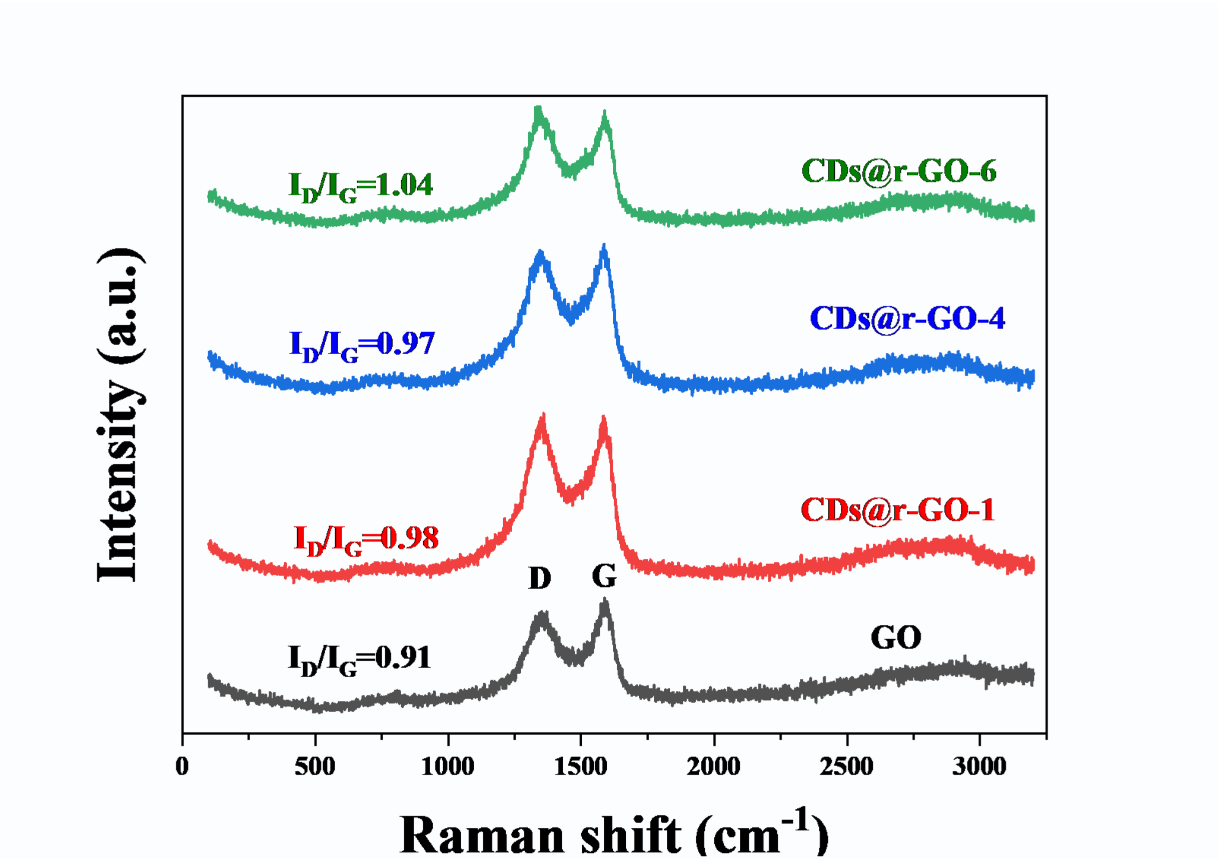


**Figure S5**. The Raman spectra of GO, CDs@r-GO-1, CDs@r-GO-4 and CDs@r-GO-6.

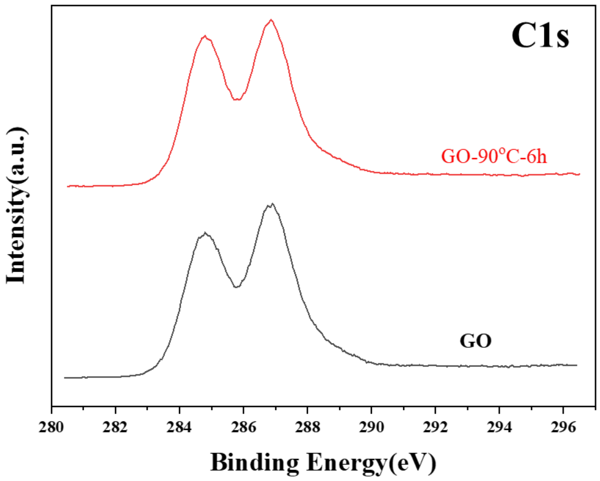


**Figure S6.** XPS C1s spectra of the GO and GO-90°C-6h.

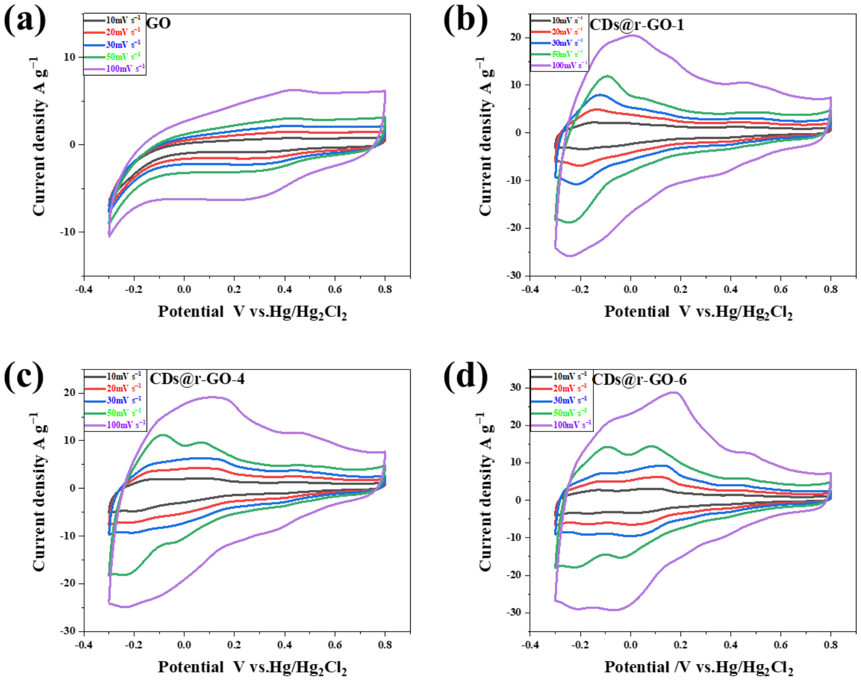


**Figure S7.** Cyclic voltammetry curves of **(a)** r-GO, **(b)** CDs@r-GO-1, **(c)** CDs@r-GO-4 and **(d)** CDs@r-GO-6 in 1 M $H_2SO_4$ electrolyte with scan rates ranging from 10 to 100 mV $s^{-1}$.

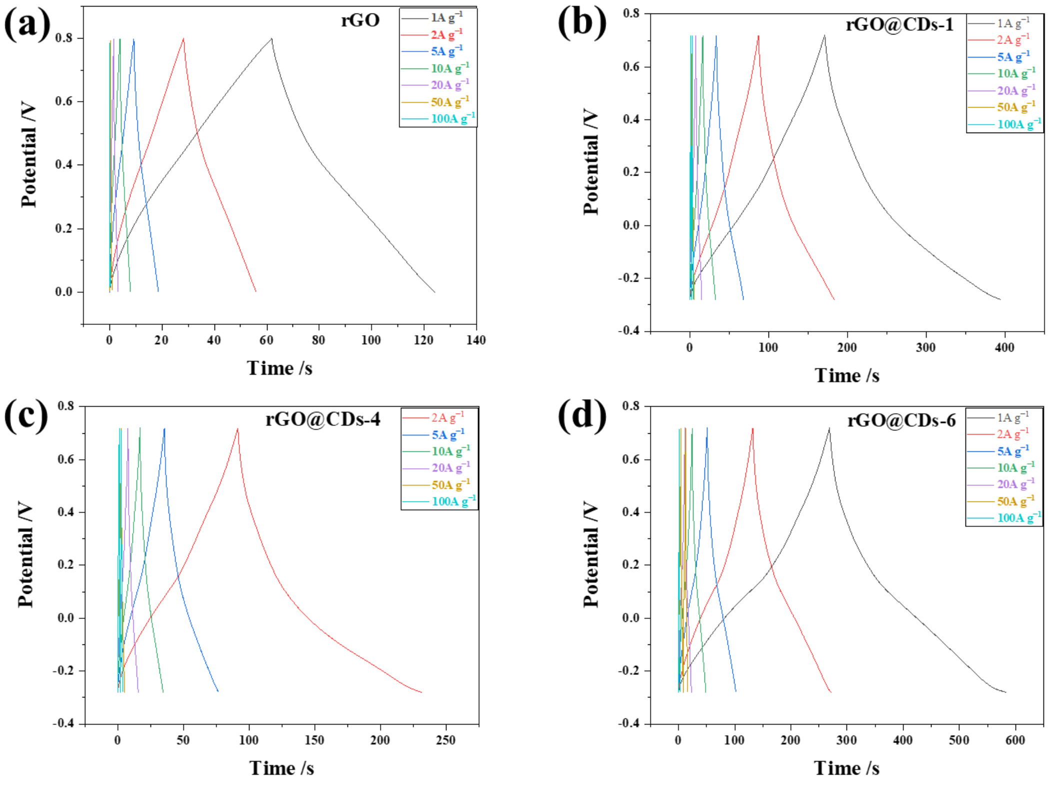


**Figure S8.** Constant current charge and discharge curve of **(a)** r-GO, **(b)** CDs@r-GO-1, **(c)** CDs@r-GO-4, and **(d)** CDs@r-GO-6 at current density of 1-100A $g^{-1}$.

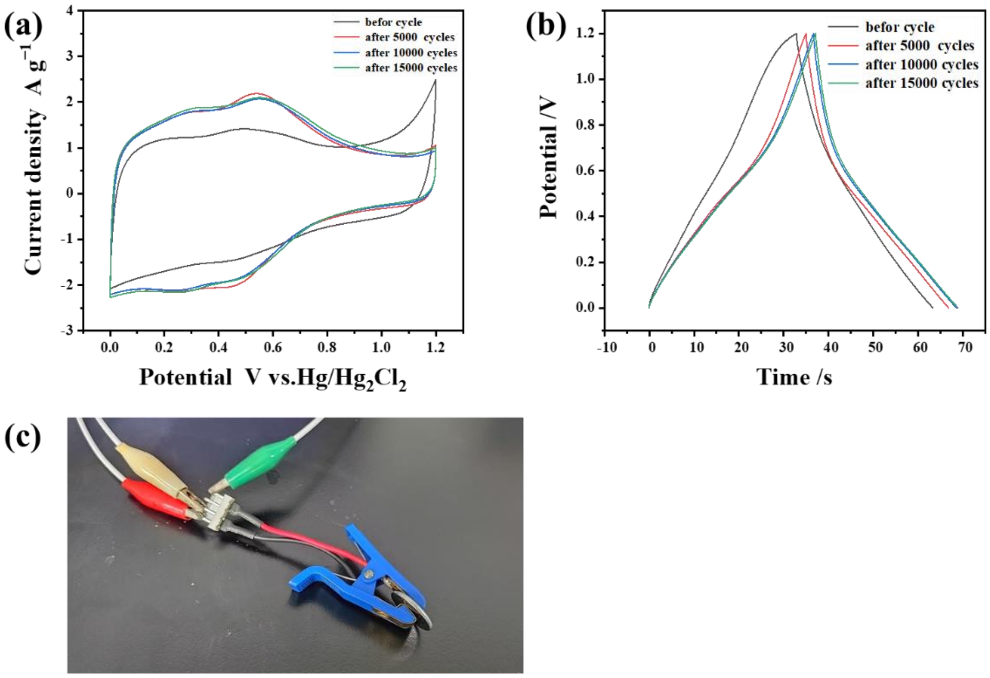


**Figure S9. (a)** Cyclic voltammetry curve of the device at a scan rate of 50 mV $s^{-1}$. **(b)** Constant current charge and discharge curve of the device at current density of 1 A $g^{-1}$. **(c)** The photo of the device fabricated.

**Table S1**. Elemental contents obtained by XPS test

| **Samples** | **C (wt.%)** | **O (wt.%)** | **N (wt.%)** |
|---|---|---|---|
| GO | 72.3 | 24.4 | 2.6 |
| GO-90$^{o}$C-6h | 72.9 | 23.6 | 3.5 |
| CDs | 46.0 | 29.5 | 14.5 |

**Table S2**. Comparison with data of electrode materials in the literature

| Electrode material | Electrolyte | Current density (A/g) | Specific capacitance (F/g) | Rate capability |
|---|---|---|---|---|
| 3D-graphene hydrogel | 1M $H_2SO_4$ | 1.0 | 186.0 | (1-20 A/g) 70% |
| Graphene hydrogel | 1M $Na_2SO_4$ | 1.0 | 157.7 | (1-10 A/g) 58.3% |
| AAQ (*2-Aminoanthraquinone*) modified SGH (self-assembled graphene hydrogel) | 1M $H_2SO_4$ | 0.3 | 258.0 | (0.3-90 A/g) 58.13% |
| Nitrogen and fluorine co-doped holey graphene hydrogel | 6M KOH | 1.0 | 345.4 | (1-100 A/g) 57.29% |
| Reduced graphene hydrogel | 6M KOH | 0.5 | 203.9 | (1-100 A/g) 59.38% |
| Reduced graphene oxide hydrogel/Carbon dots | 1M $H_2SO_4$-PVA | 1.0 | 264.0 | (1-10 A/g) 79.54% |
| Reduced graphene oxide/Carbon dots | 1M $H_2SO_4$ | 0.2 | 278.0/227.0 | (0.2-100 A/g) 60% |
| N-andS-codoped graphene hydrogel | 6M KOH | 1.0 | 320.0 | (1-10 A/g) 68.75% |
| Graphene hydrogel-graphene quantum dots | 1M $H_2SO_4$ | 0.5 | 451.7 | (1-20 A/g) 59.86% |
| Graphene quantum dots-3D graphene | 1M KOH | 1.17 | 242.0 | (1-25 A/g) 26.8% |
| N,O co-doped CDs-graphene hydrogel | 1M $H_2SO_4$ | 1.0 | 335.1 | (1-20 A/g) 67.16% |

| | | | | |
|---|---|---|---|---|
| g-C3N4/graphene hydrogel nanocomposites | 1M KOH-PVA | 0.2 | 243.2 | None |
| **This work** | 1M $H_2SO_4$ | 1.0 | 290.0 | (1-100 A/g) 78.94% |
